# Induction of MiR-21 by Stereotactic Body Radiotherapy Contributes to the Pulmonary Fibrotic Response

**DOI:** 10.1371/journal.pone.0154942

**Published:** 2016-05-12

**Authors:** Ok-Seon Kwon, Keun-Tae Kim, Eunioo Lee, Myoungjae Kim, Seo-Hyun Choi, Henghong Li, Albert J. Fornace, Jae-Ho Cho, Yun-Sil Lee, Ji-Seon Lee, Yoon-Jin Lee, Hyuk-Jin Cha

**Affiliations:** 1 Department of Life Sciences, Sogang University, Seoul, Korea; 2 College of Pharmacy and Wonkwang Oriental Medicines Research Institute, Wonkwang University, Jeonbuk, Korea; 3 Laboratory of Radiation Effect, Division of Radiation effect, Korea Institute of Radiological and Medical Sciences, Seoul, Korea; 4 Department of Biochemistry and Molecular and Cellular Biology, Lombardi Comprehensive Cancer Center, Georgetown University, Washington, D.C., United States of America; 5 Department of Radiation Oncology, Severance Hospital, Yonsei University, Seoul, Korea; 6 School of Pharmacy, Ewha University Seoul, Korea; 7 Burn Institute, Hangang Sacred Heart Hospital, College of Medicine, Hallym University, Seoul, Korea; University of Alabama at Birmingham, UNITED STATES

## Abstract

Radiation-induced lung fibrosis, the most serious effect of lung cancer radiotherapy on normal tissue, remains a major technical obstacle to the broader application of radiotherapy to patients with lung cancer. This study describes the use of an image-guided irradiation system in mice mimicking stereotactic body radiotherapy (SBRT) to examine the molecular features of chronic fibrotic response after radiation injury. MicroRNA (miR) array analysis of injured pulmonary tissue identified a set of miRs whose expression was significantly increased in damaged lung tissue. In particular, miR-21 expression was increased at the radiation injury site, concurrent with collagen deposition. Although the inhibition of miR-21 by its specific inhibitor anti-miR-21 only marginally affected endothelial-mesenchymal transition (EndMT) in lung endothelial cells, this inhibition significantly reduced collagen synthesis in lung fibroblasts. Furthermore, ectopic expression of miR-21 was sufficient to promote a fibrotic response in lung fibroblasts, enhancing Smad2 phosphorylation concurrent with Smad7 downregulation. These findings indicate that the induction of miR-21 expression is responsible for fibrotic responses observed in mesenchymal cells at the injury site through the potentiation of TGF-β signaling. Local targeting of miR-21 at the injured area could have potential therapeutic utility in mitigating radiation-induced lung fibrosis.

## Introduction

Radiation pneumonitis is one of the most frequent post-treatment complications of stereotactic body radiotherapy (SBRT), with symptoms ranging from mild to critical. In particular, radiation-induced lung fibrosis (RILF), which is characterized by the recruitment of fibroblasts, myofibroblasts, and leukocytes, resulting in the accumulation of extracellular matrix proteins, such as collagens, is a severe complication that may be life-threatening due to the impairment of normal breathing [1–3]. At present, steroid anti-inflammatory drugs are the only medical option for patients with a high risk of RILF onset prior to augmentation of the pathogenic response. However, no early diagnostic markers of RILF have yet been identified. Thus, understanding the molecular mechanism underlying the onset of RILF and identifying possible early markers for this condition may help in the development of alternative treatment strategies [4–6].

The onset of RILF is a complicated process that involves various cell types [1]. Because a marked increase in fibroblast accumulation, followed by collagen production, is the most common event in RILF, myofibroblasts and fibroblasts derived from various sources play key roles in pathophysiological fibrotic responses. Thus, novel strategies to control fibroblast accumulation or collagen production may be therapeutic in patients with RILF. Endothelial cells (ECs) injured by ionizing radiation (IR) or bleomycin have been found to acquire the cellular properties of fibroblasts in a process termed endothelial-to-mesenchymal transition (EndMT) [7–9]. EndMT is characterized by the expression of mesenchymal markers, including alpha-smooth muscle actin (α-SMA), vimentin, and fibroblast-specific protein-1 (FSP-1), and the increased production of collagens, particularly types I and III, with the latter responsible for the fibrotic responses of the ECs.

MicroRNAs (miRs) are small noncoding RNAs that bind to specific mRNA sequences and suppress target protein(s) expression by inhibiting translation or degrading mRNAs [10]. The expression of miRs has been shown to be upregulated or downregulated under various pathophysiological conditions [10]. The levels of expression of certain miRs, which can be readily determined in bodily fluids such as blood, serum, urine and saliva, have been extensively studied because of their possible applications as biomarkers for the early detection of several specific diseases [11–13]. Moreover, because the aberrant expression of certain miRs is closely associated with target protein expression, these miRs are responsible for the onset of pathophysiological responses in a disease condition. For example, miR-21, whose expression is upregulated in chronic inflammatory conditions or by transforming growth factor β (TGF-β), has been shown to potentiate TGF-β responses by downregulating the level of the inhibitory Smad, Smad7 [14]. In particular, miR-21 levels are significantly increased in the lungs of bleomycin- and TGF-β-treated mice and are involved in the pro-fibrogenic responses of TGF-β through the suppression of Smad7 [15]. These findings suggested that miR-21 may be a potential molecular target for modulating pulmonary fibrosis [15].

This study used an image-guided irradiation system in mice to mimic the radiation lung damage caused by SBRT and examined the molecular events occurring in the damaged lungs. Analysis of miR arrays showed that miR-21 was induced in damaged areas of the lungs and that miR-21 expression, along with the suppression of Smad7, contributed to collagen production by fibroblast-like cells after radiation damage. These findings suggest that a strategy to inhibit miR-21 induction after SBRT would be therapeutically effective in preventing the onset of RILF and RILF-associated pathological symptoms.

## Materials and Methods

### Reagents and cell culture

Primary antibodies against, ICAM-1 (sc-8439), p21 (sc-397), col3A1 (sc-28888), β-actin (sc-47778) and α-tubulin (sc-8035) were obtained from Santa Cruz Biotechnology (Santa Cruz, CA, USA). Primary antibodies against alpha-smooth muscle actin (a-SMA) (#a5228) were purchased from Sigma (St Louis, MO, USA). Human pulmonary endothelial cells were maintained in endothelial cell growth medium MV2 (c-22022, promocell, Heidelberg, Germany) with 0.1% gentamicin (Sigma) at 37°C in a humidified 5% CO_2_ incubator.

### Animal models and irradiation

All studies with mice were approved by the Yonsei University Medical School Animal Care and Use committee. As described previously [16], C57BL/6 male mice (8 weeks) were used for radiation-induced lung fibrosis research. To mimic SBRT conditions, 3-mm collimator to administrate a 90Gy of dose of radiation to the left lung was used for introducing the radiation damage. Right lung at each mouse was used for the control. Radiation was delivered with an X-RAD 320 (Precision, North Branford, CT, USA), equipped with a collimator system composed of 5-cm-thick copper to produce focal radiation beams. For positioning the radiated area, the image-guided devise with a motorized, 2D moving stage, a fluorescent screen (Kodak Min-R 2000; Carestream Health Inc., Rochester, NY, USA), and a charge-coupled device (CCD) camera (650D; Canon Inc., Tokyo, Japan) controlled by a mobile computer was used. Prior to irradiation, the mice were anesthetized with an intraperitoneally administered mixture of 30 mg/kg of zoletil and 10 mg/kg of rompun. The four legs of the mice were additionally fixed with adhesive tape. At 1, 2, 3 and 4 weeks after radiation, the mice were sacrificed. Four mice were allocated per group, and the experiment was repeated twice. The condition of animals after irradiation was monitored daily basis. The animals after irradiation lived normally until they were scarified at 3 or 4 weeks. The typical symptoms of high radiation exposure were observed such as hair loss and dermonecrosis at 2–3 weeks after irradiation. For lung harvesting, the mice were anesthetized with an intraperitoneally administered mixture of 30 mg/kg of Zoletil (tiletamine 25mg/kg with zolazepam 25mg/kg) and 10 mg/kg of Rompun (xylazine 10mg/kg), which produces general anesthesia in animal for 2–3 hours.

### MicroRNA microarray and analysis

Total RNA of each sample was isolated by using the Trizol reagent (Life technologies, Inc., Carlsbad, CA) according to the manufacturer’s instructions. For control and test RNAs, the synthesis of target miR probes and hybridization were performed using Agilent’s miR Labeling Reagent and Hybridization kit (Agilent Technologies, Palo Alto, CA) according to the manufacturer’s instructions. All data normalization and selection of fold-changed probes were performed using GeneSpringGX 7.3 (Agilent Technologies). We performed data transformation (set measurements less than 0.01 to 0.01) and per chip (normalize to 75^th^ percentile) normalization.

### TaqMan Real-time PCR microRNA assay

Total RNA was extracted with Trizol reagent (Invitrogen) and miRs were detected via a TaqMan Real-time PCR microRNA assay (Applied Biosystems, Carlsbad, CA). In order to quantify the different expressions of specific miR, we analyzed the expression of miRs (miR-21; 000397, U6; 001937; Ambion, Austin, Tx) in the total RNA extracted from various tissue samples and cells via real-time PCR using a TaqMan microRNA Assay kit (Applied Biosystems, Carlsbad, CA).

### RNA isolation and quantitative Real-Time PCR Analysis

Total RNA of each sample was isolated by using the Trizol reagent (Life technologies, Inc., Carlsbad, CA) according to the manufacturer’s instructions. Gene-specific primers are listed as follows: mouse col1A2 (forward: 5’-TGGTCTTACTGGGAACTTTGCTGC-3’, reverse: 5’-ACCCTGTGGTCCAACGACTCCTCTC-3’), housekeeping gene mouse GAPDH (forward: 5’-TATGGAAAGCTGTGGCCTGG-3’, reverse: 5’-CAGATGCCTGCTTCACCA CCTTC-3’), human ICAM-1 (forward: 5’-CGTGGGGAGAAGGAGCTGAA-3’, reverse: 5’-CAGTGCGGCACGAGAAATTG-3’), human aSMA (forward: 5'-CATCACCAACTGGGA CGACATGGAA-3', reverse: 5'-GCATAGCCCTCATAGATGGGGACATTG-3'), human fibronectin (FN) (forward: 5'-GTGTTGGGAATGGTCGTGGGGAATG-3', reverse: 5'-CCA ATGCCACGGCCATAGCAGTAGC-3'), human col1A2 (forward: 5'-GGTGGTGGTTATG ACTTTGG-3', reverse: 5'-TCTGGGTGGCTGAGTCTCAA-3'), human col3A1 (forward: 5'-GCTCTGCTTCATCCCACTATTA-3', reverse: 5'-AACATTCTCCAAATG GAATT-3') and housekeeping gene human b-actin (forward: 5’-GTCCTCTCCCAAGTCCACAC-3’, reverse: 5’-GGGAGACCAAAAGCCTTCAT-3’). All amplifications were conducted in a pre-mixture (20 ml) containing 500 nmol/l of gene-specific primers, and 6 ml of template, under the following conditions: denaturation at 95°C for 1 min, followed by 40 cycles of 95°C for 15 sec, 58°C for 15 sec, and 72°C for 15 sec, with a final extension at 72°C for 5 min. For PCR validation, amplified products were separated on 2% agarose gels and visualized by ethidium bromide staining. The reactions were carried out in Roche LC480.

### In situ hybridization

miRCURY LNA^™^ microRNA ISH Optimization Kit (FFPE) was purchased from Exiqon (Vedbaek, Denmark). In situ hybridization was performed with 3μM-thick mouse lung tissues and all procedure was proceeded in RNase-free environment. ISH probe for miR-21 and scramble control were 5’ and 3’- digoxigenin (DIG) labeled. U6 was used as positive control and labeled at 5’-end only. The experiment was performed according to a company’s protocol. Briefly, tissue slides were deparaffinized and treated with proteinase-K. After that probe hybridization, stringent washes, blocking steps were performed. Finally, slides were treated with anti-DIG reagent, NBT/BCIP substrate (nitro-blue tetrazolium and 5-bromo-4-chloro-3'-indolyphosphate), and nuclear counter staining. The section was mounted with mounting medium and analyzed under the microscopy. The pixel density of miR-21 positive area was determined by ImageJ (https://imagej.nih.gov/ij/) software package.

### Statistics

The graphical data were presented as mean ± S.E.M. Statistical significance among the three groups and between groups was determined using one-way or two-way analysis of variance (ANOVA) following Bonferroni post-test and Student’s t-test respectively. Significance was assumed for p < 0.05 (*), p < 0.01 (**), p < 0.001 (***), p < 0.0001 (****).

## Results

### Local IR exposure mimicking SBRT induces lung fibrosis

**Fig 1:**
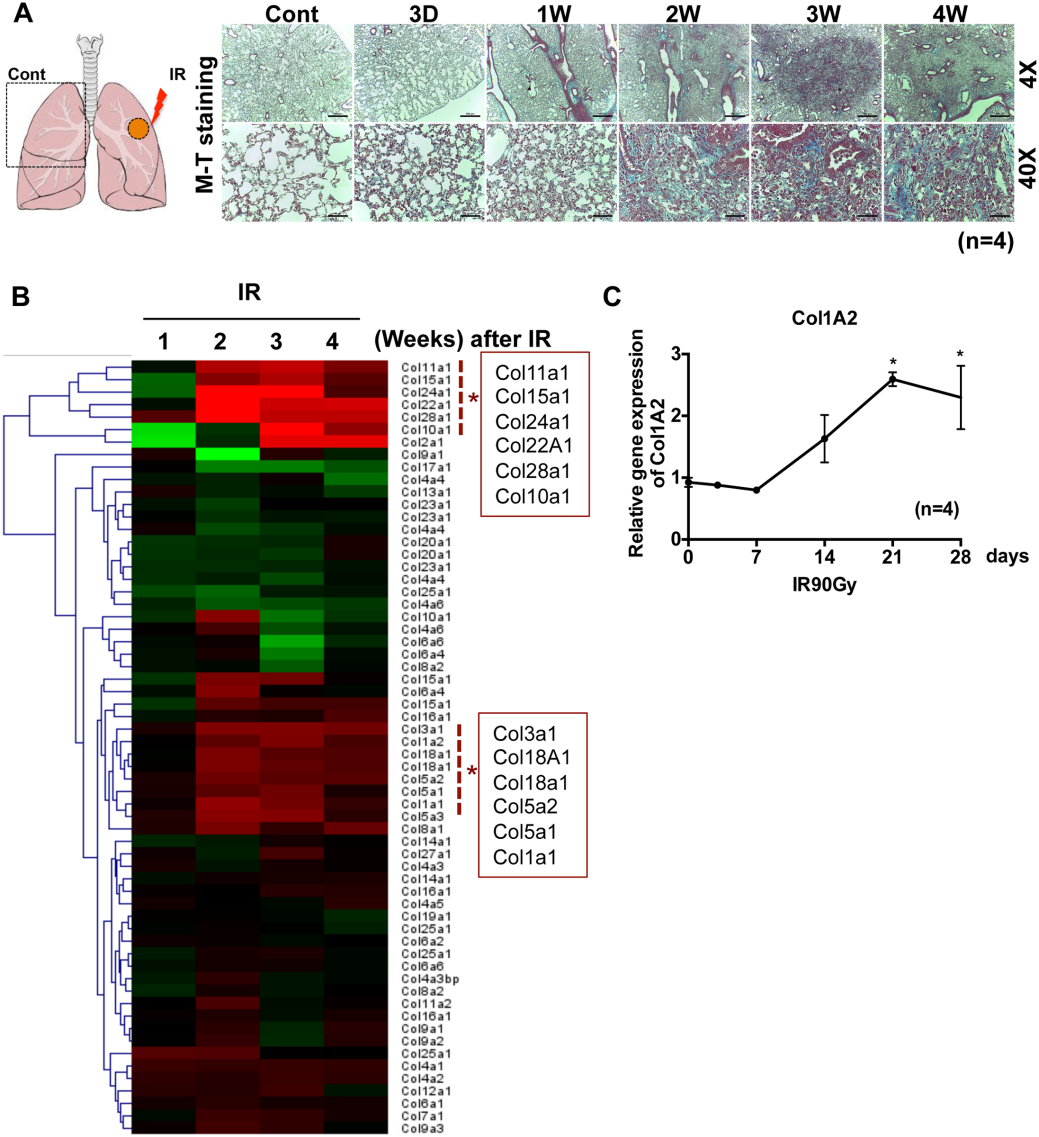
Local IR exposure mimicking SBRT induces lung fibrosis. (A) Schematic diagram of IR exposure on mouse lung (left panel). Lung tissue isolated from the left lung was stained with Masson’s Trichrome at indicative time after 90Gy exposure (right panel). The representative images were shown (right panel) (n = 4). (B) Heat-map of collagen subtypes from the IR lung tissue (left side lung) compared to the control (right side of lung) (n = 4) (red dotted line and asterisk for significantly upregulated collagen subtypes after IR) (C) The mRNA level of Col1A2 was determined by real-time PCR at indicative time after 90Gy exposure (n = 4, * p<0.05).

The left lungs of mice were exposed to a single IR dose of 90 Gy (X-rays), as described previously [16]. All mice were viable for at least 4 weeks, at which time they were sacrificed and their lungs were harvested. Collagen deposition markedly increased in damaged areas of the left lungs, beginning 2 weeks after IR exposure, suggesting the development of RILF (Fig 1A). The molecular events occurring during RILF were examined by microarray analysis comparing the left and right lungs at each time point (Fig 1A, left panel). Gradual increases in the levels of several collagen subtypes (11A1, 15A1, 24A1, 22A1, 28A1, 10A1, 2A1, 1A1, 1A2 and 3A1) were apparent, beginning 2 weeks after IR exposure, with their levels of expression sustained until sacrifice at 4 weeks (Fig 1B, red dotted box). The gradual deposition of collagens, particularly types Col1A2, Col1A1, Col3A1, Col11A1 and Col15A1, which are commonly increased during fibrosis, beginning 2 weeks after IR exposure, was confirmed by real-time PCR analysis (Fig 1C and S1 Fig).

### Upregulation of miR-21 in IR-damaged lung tissue

**Fig 2:**
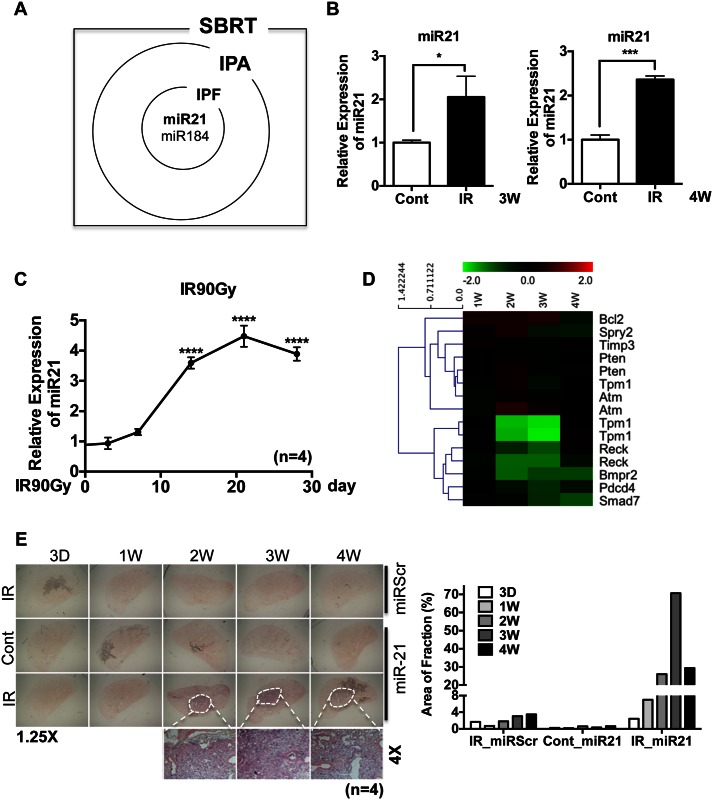
Upregulation of miR-21 in IR-damaged lung tissue. (A) Venn diagram for upregulated miRs from the microRNA array on SBRT tissue (SBRT), Ingenuity pathway analysis (IPA) and Idiopathic pulmonary fibrosis (IPF). (B) miR-21 expression in IR treated lung (left side) compared to control (right side) in 3 week and 4 weeks after IR treatment. The average ct values for miR-21 of 3 week and 4 week are 18.6 and 17.43 respectively. (n = 3, p<0.05 (*), p<0.001 (***)) (C) Level of miR-21 in the left side of lung compared to the control (right side of lung) at indicative time after IR was determined by taqman miR real-time PCR using U6 snRNA for an internal control. (n = 4, p<0.0001 (****)) (D) Heat-map of miR-21 target genes from the IR lung tissue compared with control for indicated time point. (E) In situ hybridization for miR-21 with either scramble probes (miRScr, top panels) or miR-21 (miR-21, middle and bottom panels) in right side of lung (Cont) or left side of lung (IR) (left panel). Pixel density of miR-21 positive area was quantified and shown as a bar graph (right panel).

Recently, miRs whose expression is upregulated in various fibrotic diseases of the skin, kidney, liver and lung were shown to contribute to fibrotic responses [11, 17, 18], suggesting that selective modulation of miRs may be a promising non-steroidal approach to treat fibrosis. miR arrays were prepared from left and right lung tissue samples 3 and 4 weeks after exposing left lungs to IR to identify any miR that may be associated with RILF. Scatter plots of miR expression profiles (cut-off score = 2) at 3 and 4 weeks revealed that the levels of expression of several miRs were significantly altered by IR exposure (S2 Fig). The miRs significantly altered at both time points were subjected to Ingenuity Pathway Analysis (IPA) and mapped to the Ingenuity knowledge database, which incorporates microRNA-mRNA interactions from the databases TarBase, miRecords, and TargetScan, and includes associations of miRs with diseases based on published literature. This analysis indicated that miR-21 and miR-184, which are upregulated in idiopathic pulmonary fibrosis (IPF) [19, 20], were significantly upregulated in IR-injured mouse lung tissue (Fig 2A).

Because miR-21 is involved in fibrosis of multiple tissues, including the lungs [15] and kidneys [21], we focused on miR-21 in IR-injured mouse lungs. Several mRNAs, including those encoding p53, have been shown to interact with miR-21 (S1B Fig) [22, 23]. Real-time RT-PCR analysis confirmed that miR-21 was induced in IR-damaged lung tissue after 3 and 4 weeks (Fig 2B). More importantly, the increased expression of miR-21 in damaged lungs (Fig 2C) strongly correlated with the level of collagen expression (Fig 1B and 1C), a phenotype typical of fibrosis. Monitoring the levels of nine genes, whose expression is regulated by miR-21, in microarrays, showed that several of these target genes, including Spry2, Tpm1, Reck, Bmpr2, Pdcd4 and Smad7 (http://www.genome.jp/kegg-bin/show_pathway?hsa05206), were significantly downregulated at 3 and 4 weeks, at the time of miR-21 induction (Fig 2D). Finally, *in situ* hybridization showed that miR-21 was markedly increased only at IR-damaged areas (Fig 2E, white circles), suggesting that the local induction of miR-21 in these IR-damaged lung areas may contribute to the progression of pulmonary fibrosis.

### IR promotes EndMT in lung ECs concurrent with miR-21 induction

**Fig 3:**
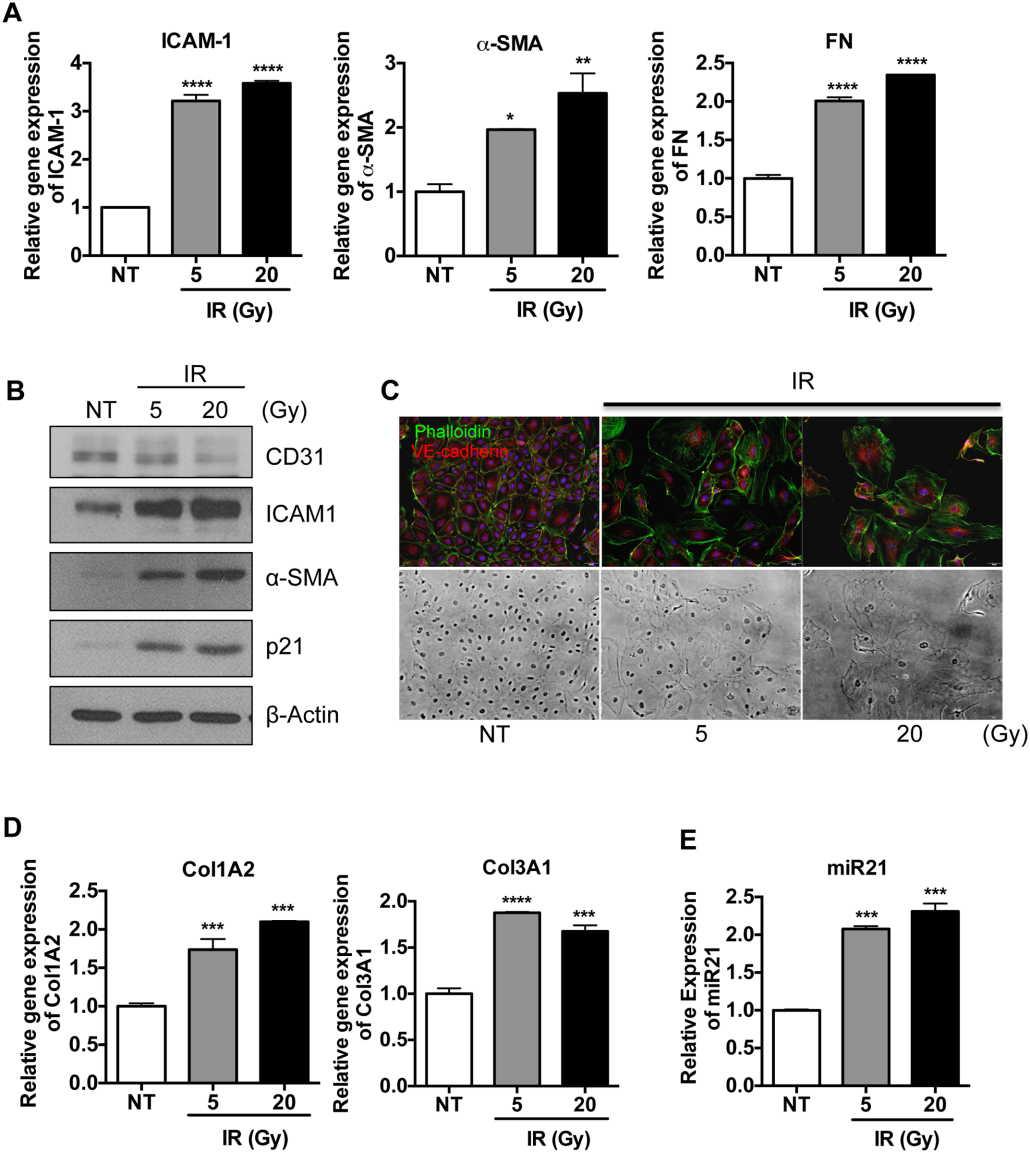
IR promotes EndMT in lung ECs concurrent with miR-21 induction HPECs were exposed with either 5 or 20 Gy of X-ray and were harvested after 3 days. (A) The mRNA levels of ICAM-1 (left panel), α-SMA (middle panel), FN (right panel) were determined by real-time PCR analysis. β-actin for an equal loading control. Each data represents one of the experiments conducted three times with duplicate samples. (B) The protein level of CD31, ICAM-1, α-SMA and p21 were determined by immunoblotting analysis. α-tubulin was used for loading control (C) HPECs was immunostained with phalloidin (green) and α-VE-cadherin antibody (red). DAPI was used for nuclear counterstaining (blue). Corresponding bright-field microscopic images were displayed in the bottom panel. (D) The mRNA levels of collagen genes (Col1A2 and Col3A1) were determined by real-time PCR analysis. β-actin for a loading control (E) The level of miR-21 of HPECs was determined via taqman miR real-time PCR using U6 snRNA for an internal control. (D-E) Each result reproduced four times and the representative results were shown. ((p < 0.05 (*), p < 0.01 (**), p < 0.001 (***) and p < 0.0001 (****)).

Recent evidence has revealed that fibroblast-like cells, which are responsible for the pathological symptoms of RILF due to the hyper-production of collagens, are derived from pulmonary ECs through EndMT following radiation injury [1, 24]. Therefore, we tested whether miR-21 induction in damaged lung tissue (Fig 2) was associated with EndMT. Human pulmonary microvascular ECs (HPECs) were exposed to IR, inducing EndMT [25], and miR-21 expression was evaluated. IR damage with 5 Gy and 20 Gy doses of X-rays induced the expression of intercellular adhesion molecule 1 (ICAM-1), a marker of IR-induced damage [26, 27], alpha-smooth muscle actin (α-SMA), and fibronectin (FN), all of which are markers of mesenchymal transition (Fig 3A). The reduction in expression of CD31 antigen and the increase in expression of α-SMA in IR-exposed HPECs, accompanied by IR dose-dependent ICAM-1 and p21CIP1 expression, indicated that these HPECs had undergone EndMT (Fig 3B). The cell surface of control HPECs showed predominant arrangements of phalloidin-labeled actin, in tight association with cell-to-cell adhesions and clear morphological changes (Fig 3C) [28]. After IR exposure, these HPECs underwent morphological changes typical of EndMT, such as increased actin reorganization and a loss of VE-cadherin at the cell surface, concurrent with cell-to cell adhesion (Fig 3C) [29]. To confirm that HPECs after EndMT (M-HPECs) acquire mesenchymal properties, collagen production by M-HPECs was determined by real-time RT-PCR. These cells showed marked upregulation of expression of mRNAs encoding collagen types I α2 (Col1A2) and III α1 (Col3A1) (Fig 3D), both of which are also clearly increased in IR-damaged lung tissues (Fig 1B). Moreover, ColA2, Col3A1 and FN mRNAs were increased after HPECs were exposed to 5Gy radiation (S3A Fig). Notably, while M-HPECs displayed mesenchymal properties (Fig 3A, 3B, 3C and 3D), their levels of miR-21 expression were also significantly upregulated, similar to findings in IR-damaged lung tissue, which indicated that miR-21 induction at IR-damaged areas of the lung may contribute to either EndMT or collagen production (Fig 3E and S3B Fig).

### miR-21 expression is dispensible for EndMT

**Fig 4:**
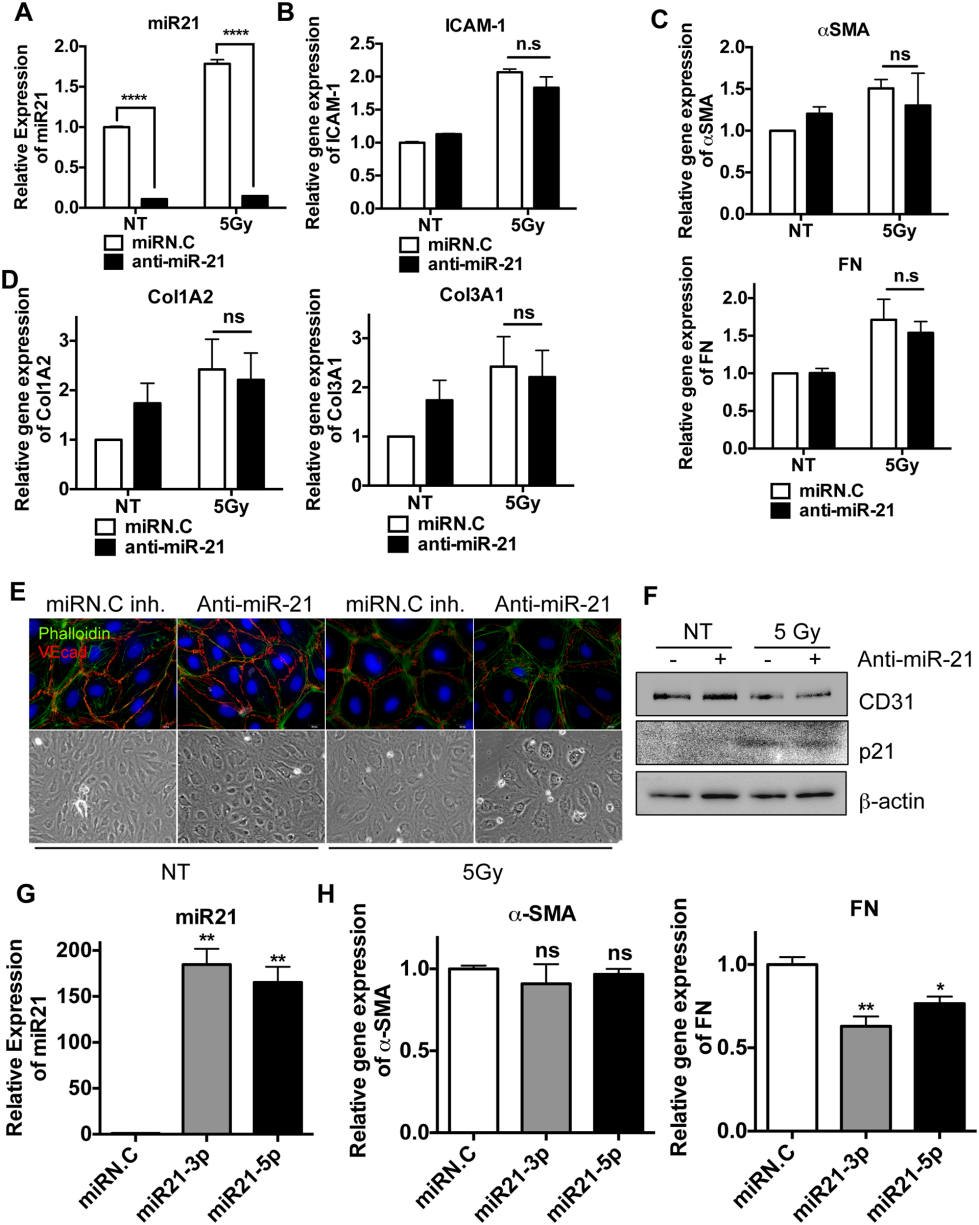
miR-21 expression is dispensible for EndMT HPECs were transfected with scramble miRs (miRN.C inh) or miR-21 inhibitor (anti-miR-21) (500nM). 24 hours after, cells were exposed to 5 Gy of X-ray (IR) and were harvested after 3 days. (A-D) Each sample was loaded as a duplicated and four independent experiments were achieved. The representative results were shown. (A) The level of miR-21 after either scramble miRs (miRN.C) or miR-21 inhibitor (anti-miR-21) transfection was determined by taqman miR realtime-PCR. U6 snRNA for equal loading control (B) The mRNA levels of ICAM-1 by real-time PCR analysis 3 days after 5 Gy of X-ray (C) The mRNA levels of α-SMA (top panel), FN (bottom panel) by real-time PCR (D) The mRNA levels of collagen subtypes (Col1A2: top panel and Col3A1: bottom panel) by real-time PCR, β-actin for a equal loading control. (E) HPECs were immunostained with phalloidin (green) and α-VE-cadherin antibody (red). DAPI was used for nuclear counterstaining (blue). Corresponding bright-field microscopic images were displayed in the bottom panel. (F) Protein level of CD31 and p21 were determined by immunoblotting analysis. β-actin was used for an equal loading control. (G-H) HPECs were transfected with 30nM of miR-21 mimics (hsa-mir-21-3p and -5p) and then were harvested at 48hr. Each sample was loaded as a duplicated and three independent experiments were accomplished. The representative results were presented. (G) The level of miR-21 level by taqman miR real-time PCR with U6 snRNA for an internal control (H) The mRNA levels of α-SMA and FN by real-time PCR, β-actin was used for an equal loading control. (ns: not significant, p < 0.05 (*), p < 0.01 (**), p < 0.001 (***) and p < 0.0001 (****))

The finding that miR-21 was induced in M-HPECs after IR exposure (Fig 3E), similar to results in IR-injured lung tissue (Fig 2), suggests that miR-21 expression in M-HPECs may be important for EndMT. This hypothesis was also supported by results showing that miR-21 contributes in part to TGF-β-induced EndMT [30]. HPECs were therefore treated with anti-sense miR-21 to inhibit miR-21 expression [15], followed by EndMT induction by exposure to IR. Despite the efficient inhibition of miR-21 by anti-sense miR-21, even after IR exposure (Fig 4A), as well as the response to radiation as determined by the induction of ICAM-1 (Fig 4B), the expression of EndMT markers, such as α-SMA, FN (Fig 4C) and ColA2 and ColA3 (Fig 4D), was not affected. Moreover, miR-21 inhibition had no effect on IR-induced morphological changes in HPECs, as shown by increases in stress actin fibers and by losses of VE-cadherin (Fig 4E). Similarly, miR-21 inhibition had no effect on IR-induced reductions in CD31 antigen levels (Fig 4F). To confirm this unexpected observation, miR-21 was overexpressed 150- to 200-fold in HPECs to determine whether this overexpression could convert endothelial-like HPECs to mesenchymal-like cells. This overexpression, however, failed to induce α-SMA (Fig 4G), while moderately reducing FN expression (Fig 4H). These results demonstrated that increased levels of miR-21 are dispensible for EndMT in HPECs.

### miR-21 contributes to collagen production in fibroblasts

**Fig 5:**
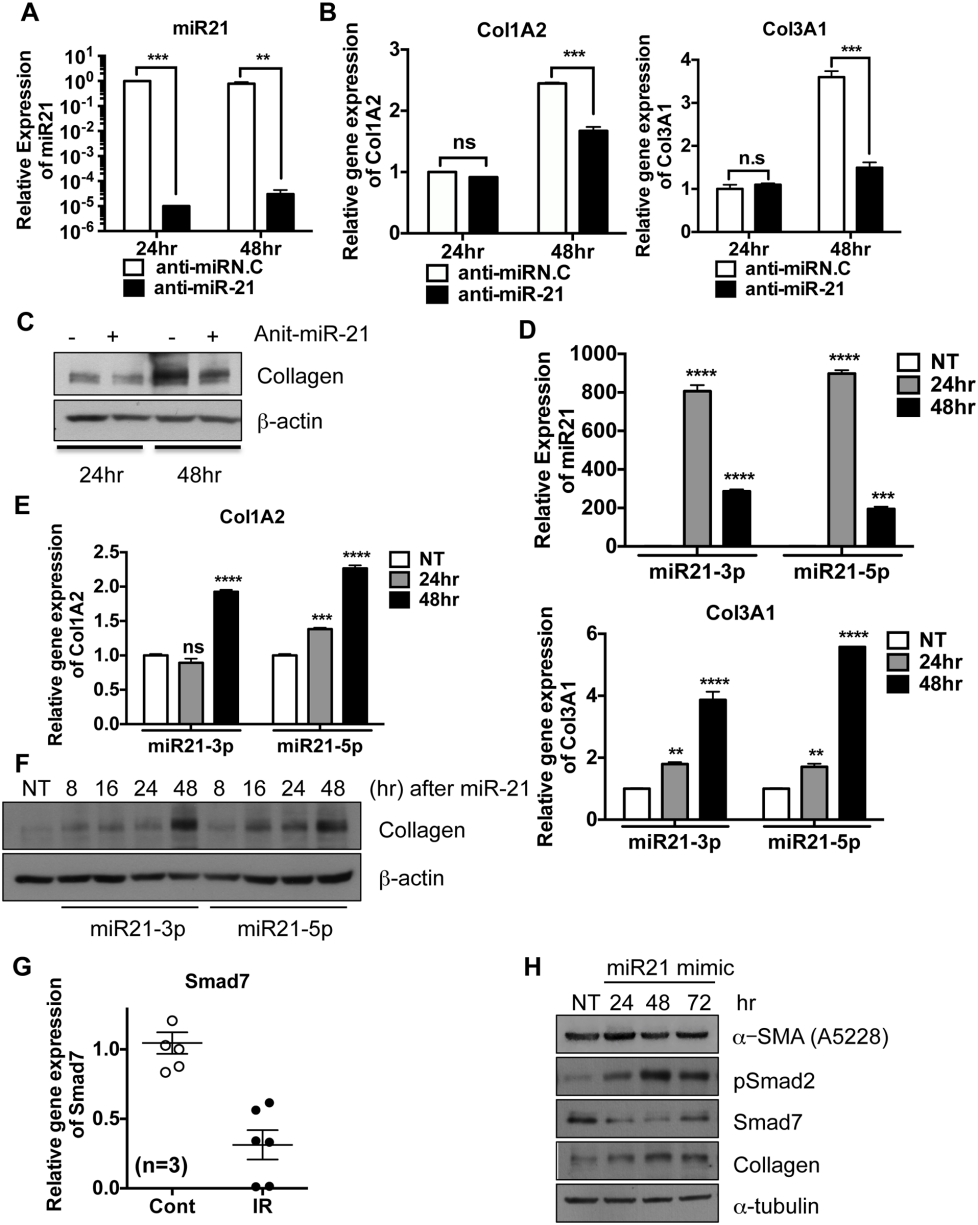
miR-21 contributes to collagen production in fibroblasts MRC-5 cells were transfected with 500nM of anti-miR-21 and then were harvested at indicative times. These data represent one of the experiments performed four times with duplicate samples (A-C). (A) The level of miR-21 level by taqman miR real-time PCR using U6 snRNA for an internal control (B) The mRNA levels of Col1A2 and Col3A1 by real-time PCR analysis and β-actin was applied for an loading control (C) The protein level of collagen 3 type α1 (Col3A1) at indicative time after miR-21 transfection (30nM) was determined by immunoblotting analysis. β-actin was used for an equal loading control. (D-E) Each sample was loaded as a duplicated and three independent experiments were achieved. The representative results were shown. (D) The level of miR-21 level by taqman miR real-time PCR using U6 snRNA for as internal control (E) The mRNA levels of Col1A2 and Col3A1 by real-time PCR, β-actin for an equal loading control (F) The level of collagen (Col3A1) was determined by immunoblotting analysis. β-actin was applied for an loading control. (G) The mRNA level of Smad7 of mouse lung at 2 weeks after radiation exposure was examined by real-time PCR analysis (n = 3). (H) The levels of α-SMA, Smad2 phosphorylation (pSmad2), Smad7 and collagen (Col3A1) were determined by immunoblotting analysis after ectopic expression of miR-21 mimic. α-tubulin for equal loading control (ns: not significant, p < 0.05 (*), p < 0.01 (**), p < 0.001 (***) and p < 0.0001 (****))

Because EndMT in HPECs does not require miR-21 overexpression (Fig 4), the role of miR-21 overexpression at IR-damaged sites remains unclear. We therefore tested whether increased miR-21 expression could augment the mesenchymal properties of fibroblast-like cells or fibroblasts after the completion of EndMT in ECs [30] by determining whether miR-21 inhibition alters collagen production in lung fibroblasts. We found that inhibition of miR-21 in MRC-5 human lung fibroblast cells by anti-sense-21 (or miR-21 inhibitor) (Fig 5A) suppressed collagen synthesis (*e*.*g*., Col1A2 and Col3A1) at both the mRNA (Fig 5B) and protein (Fig 5C) levels. Collagen synthesis, however, increased after 48 hours, when high cell confluence itself promotes collagen synthesis [31]. In contrast to findings in HPECs (Fig 4F and 4G), the expression of exogenous miR-21 in MRC-5 cells (Fig 5D) enhanced collagen synthesis at both the mRNA (Fig 5E) and protein (Fig 5F) levels in a time-dependent manner. Real-time RT-PCR analysis showed that Smad7 expression was significantly downregulated in IR-injured lung tissues (Fig 5G). Because miR-21-induced Smad7 downregulation contributes to bleomycin-induced pulmonary fibrosis (BLM fibrosis) [15], miR-21 induction in mesenchymal cells may promote the TGF-β gene response by suppressing Smad7. As predicted, expression of exogenous miR-21 alone downregulated Smad7 expression while enhancing Smad2 phosphorylation, resulting in an increase in collagen type III α from COL3A. These findings indicated that expression of exogenous miR-21 activated Smad-dependent TGF**β** signaling (S4A and S4B Fig), whereas inhibition of miR21 resulted in a Smad gene response (S4C Fig). These results clearly indicated that IR induction of miR-21 expression primarily contributes to collagen production by fibroblasts or fibroblast-like cells rather than to EndMT in ECs through the downregulation of Smad7.

## Discussion

SBRT has been used to treat patients with both primary and metastatic lung tumors [32]. Approximately 9–28% of SBRT-treated lung cancer patients, however, experience radiation-induced pneumonitis (RP), with the extent depending on the radiation dose, tumor grade, and area of normal lung tissue damages [33, 34]. Some patients with RP may progress to RILF, which may be fatal. Whereas the onset of RP occurs 1–6 months after SBRT, RILF takes months to years to develop. Thus, early diagnosis of RILF development is important for initiating anti-fibrotic treatments, such as steroids, which can effectively alleviate RILF symptoms [35–37].

miRs, particularly circulating (or serum) miRs, have been extensively studied to identify biomarkers for certain types of diseases, including cancers [38]. Identifying any miRs that are involved in RILF and that could be readily detected in patients is therefore important. This study found that miR-21 [39], which is expressed in serum, was significantly upregulated in SBRT lung tissue. Additional studies of sera from RILF patients are required to confirm that these miRs are early markers of RILF, similar to findings in patients with IPF [40]. In agreement with results showing that miR-21 is induced in BLM fibrosis [15], this study found that miR-21 levels progressively increased during collagen production at SBRT-damaged sites, accompanied by the significant downregulation of several miR-21 targets, including Smad7, suggesting that increased miR-21 contributes to fibrotic responses. Indeed, miR-21 has been shown to play a role in other fibrotic diseases, including renal [21, 41] and cardiac [42] failure. We therefore tested the ability of an anti-sense oligonucleotide to miR-21, which specifically inhibits miR-21, to delay the fibrotic response, finding that this anti-sense-miR21 was effective [15, 21, 42].

This study also showed that miR-21 induction was concurrent with collagen production at SBRT-damaged sites, promoting collagen production by mesenchymal cells but not by pulmonary ECs. Unlike results showing that miR-21 is partly responsible for TGF-β-mediated EndMT [30], this study found that miR-21 inhibition failed to rescue EndMT progression, with overexpression of exogenous miR-21 having only a slight effect on EndMT in pulmonary ECs. These data suggest that EndMT does not require miR-21 induction by SBRT. The reason for this discrepancy remains unclear, although different stimuli were used to induce EndMT (*i*.*e*., TGF-β vs IR) [30]. By contrast, the inhibition or induction of miR-21 affected fibrotic responses only in mesenchymal cells. These results suggest that miR-21, the expression of which is upregulated by a TGF-β-dependent gene response [15, 21], may potentiate fibrotic responses in myofibroblasts and fibroblasts recruited to the damaged areas through suppression of Smad7. Modulation of miR-21 expression by anti-sense-21 would still be a valid approach, as serum levels of miR-21 may increase after SBRT.

## Supporting Information

S1 Fig Induction of Collagen expression in SBRT induced lung injury. (A) The mRNA level of Collagen types (Col1A1, Col1A2, Col3A1, Col11A1 and Col15A1) of 2 and 4 weeks after 90Gy IR exposure was determined by real time PCR. Control indicated right side of the lung, which was not irradiated (n = 2). 18s ribosomal RNA was used as a loading control.
(TIF)

S2 Fig Expression of a set of miRs was altered after local exposure of IR in the lung. (A) Scatter plot of normalized intensity of miRs between control and IR damaged lung tissues at three weeks (left) and four weeks (right) (B) Heat-map of miRs (cut off range = 1.8) significantly altered in the IR damaged lung tissue compared to the control at both 3 (3W) and 4 (4W) weeks. Red dotted line and asterisk for significantly upregulated microRNAs after IR. (C) IPA of altered miRs at 3 or 4 weeks of IR damaged lung tissue
(TIF)

S3 Fig Increased levels of miR-21 are concurrent with IR-induced EndMT. HPECs were exposed with 5 Gy of X-ray and were harvested at indicative time. Each result represent one of the experiments conducted three times with duplicate samples. (A) The mRNA levels of Col1A2, Col3A1 and FN at indicative time after 5 Gy by real-time PCR analysis, β-actin for an equal loading control (B) The level of miR-21 at indicative time after 5 Gy by taqman miRNA real-time PCR using U6 snRNA for an internal control
(TIF)

S4 Fig miR-21 controls Smad dependent gene response through Smad7. (A) The level of miR-21 was determined after transfection with hsa-miR-21 mimic (miR-21). (B and C) Luciferase activity of SBE (Smad Binding Element) after ectopic expression (B) or inhibition (C) of miR-21 was determined and shown as a bar graph. (B-C) These results represented one of the experiments performed twice with triplicate samples.
(TIF)

S1 File Supplement information Supporting information includes supplement material and method (immunobloting, immunohistochemistry and dual luciferase assay) and figure legends for supplementary figure. (DOCX)
