# Correction: Induction of MiR-21 by Stereotactic Body Radiotherapy Contributes to the Pulmonary Fibrotic Response

**DOI:** 10.1371/journal.pone.0160137

**Published:** 2016-07-21

**Authors:** The PLOS ONE Staff

The third author's name is spelled incorrectly. The correct name is: Eunjoo Lee. The publisher apologizes for the error.
