# Supplementary figures and images for: Induction of MiR-21 by Stereotactic Body Radiotherapy Contributes to the Pulmonary Fibrotic Response
Source: PLoS One. 2016 May 12;11(5):e0154942. doi: 10.1371/journal.pone.0154942 (PMC4865046; doi:10.1371/journal.pone.0154942)

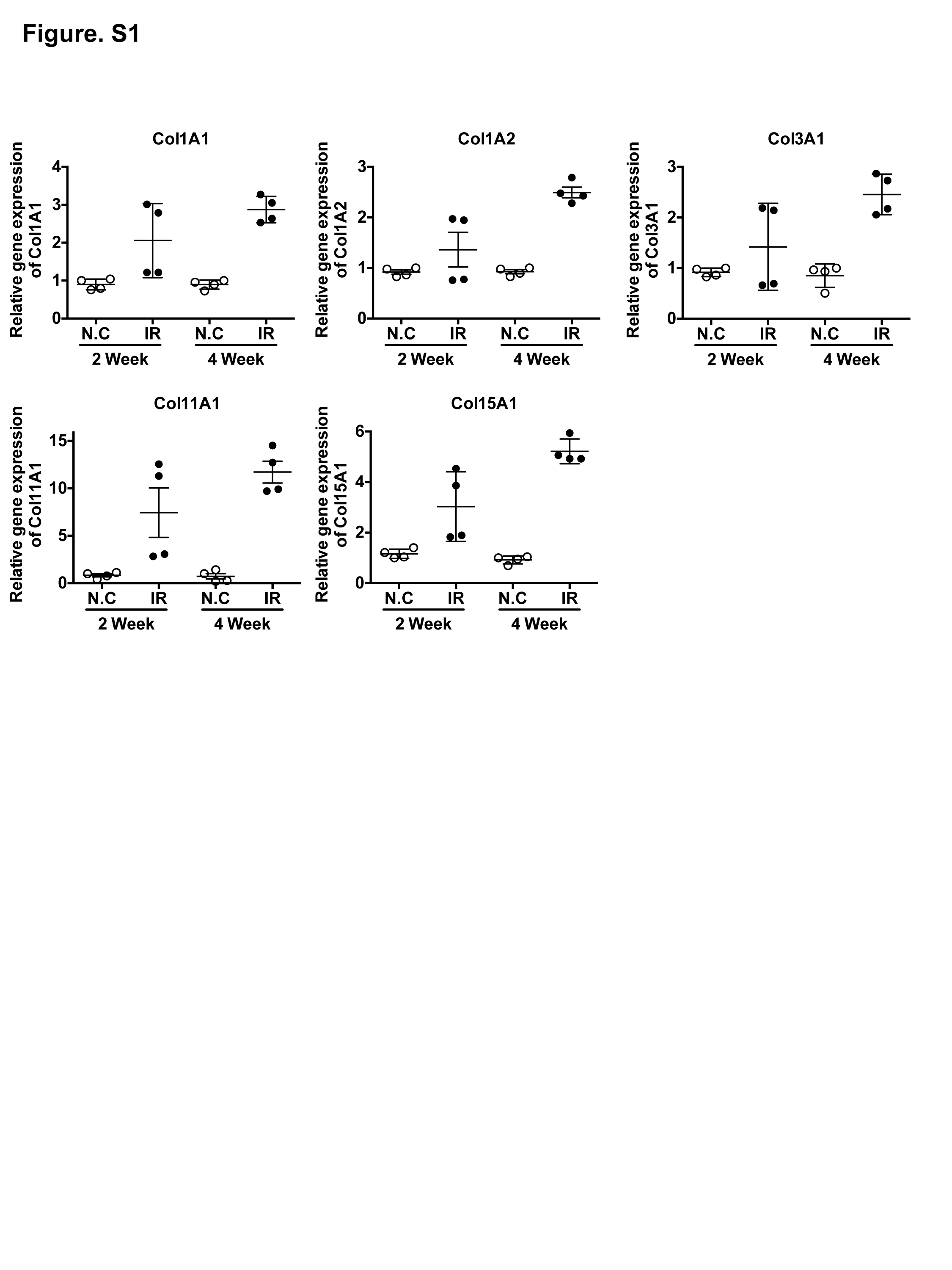

Supplement: S1 Fig — (A) The mRNA level of Collagen types (Col1A1, Col1A2, Col3A1, Col11A1 and Col15A1) of 2 and 4 weeks after 90Gy IR exposure was determined by real time PCR. Control indicated right side of the lung, which was not irradiated (n = 2). 18s ribosomal RNA was used as a loading control. (TIF) [file pone.0154942.s001.tif]

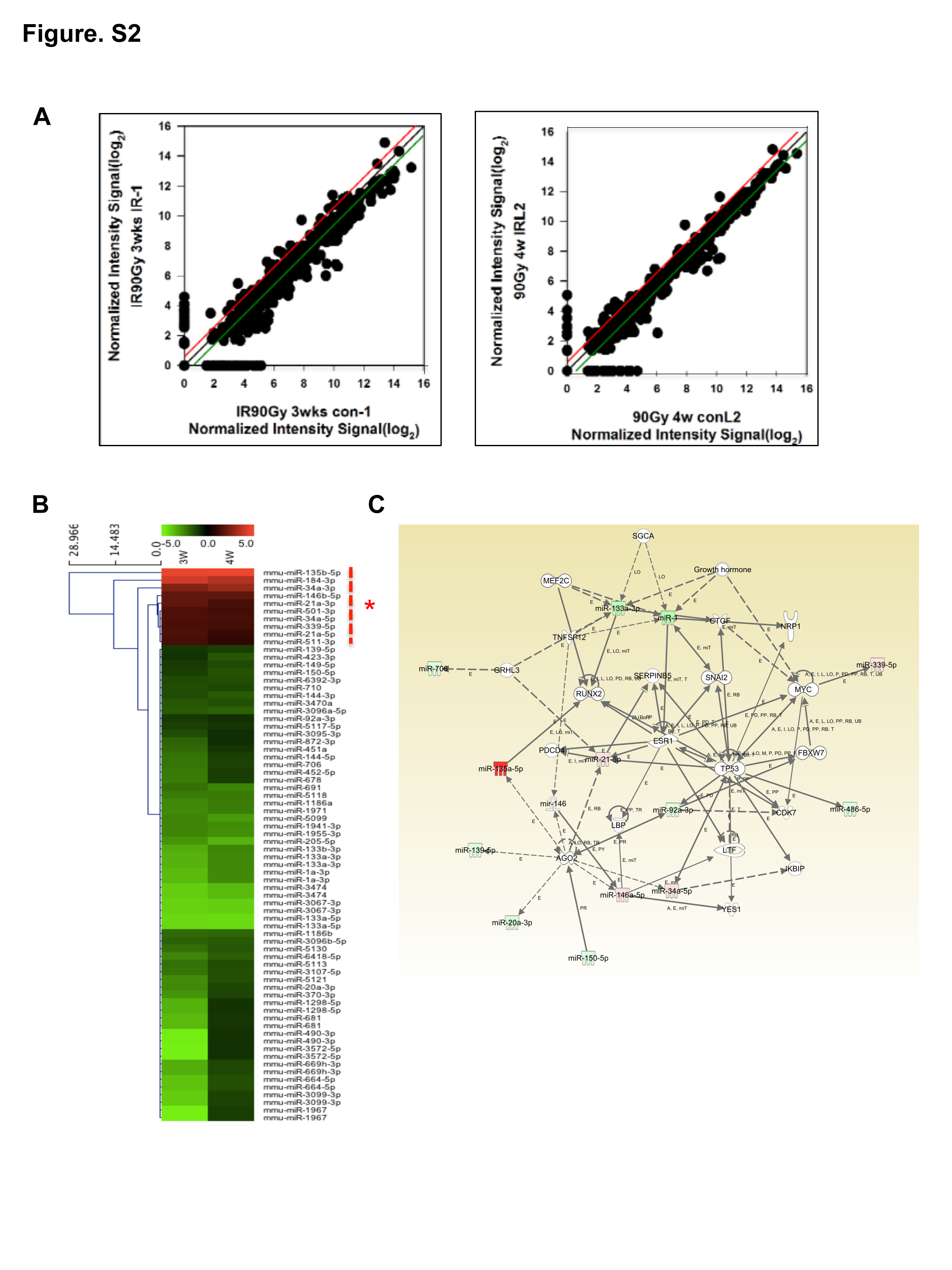

Supplement: S2 Fig — (A) Scatter plot of normalized intensity of miRs between control and IR damaged lung tissues at three weeks (left) and four weeks (right) (B) Heat-map of miRs (cut off range = 1.8) significantly altered in the IR damaged lung tissue compared to the control at both 3 (3W) and 4 (4W) weeks. Red dotted line and asterisk for significantly upregulated microRNAs after IR. (C) IPA of altered miRs at 3 or 4 weeks of IR damaged lung tissue (TIF) [file pone.0154942.s002.tif]

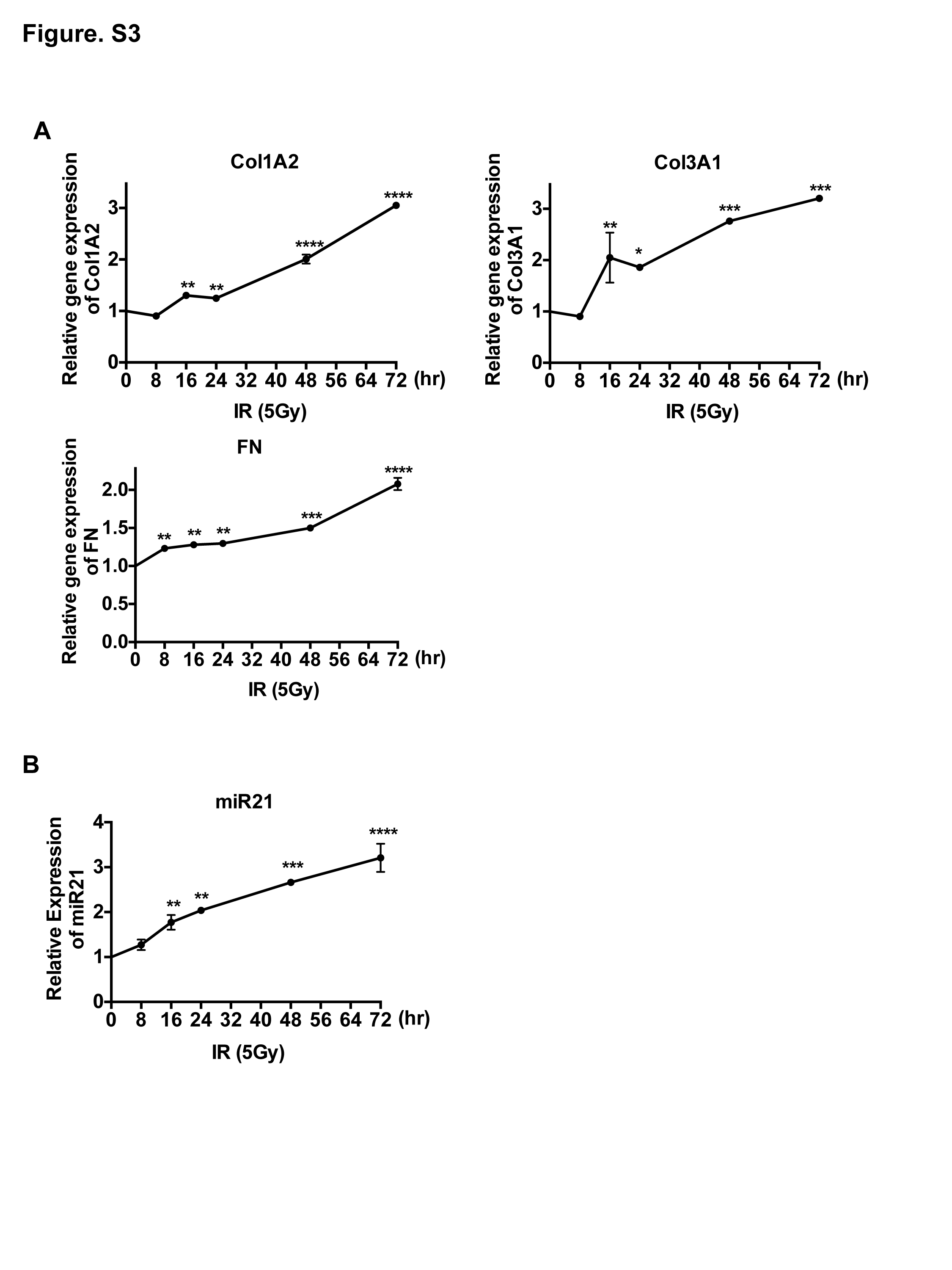

Supplement: S3 Fig — HPECs were exposed with 5 Gy of X-ray and were harvested at indicative time. Each result represent one of the experiments conducted three times with duplicate samples. (A) The mRNA levels of Col1A2, Col3A1 and FN at indicative time after 5 Gy by real-time PCR analysis, β-actin for an equal loading control (B) The level of miR-21 at indicative time after 5 Gy by taqman miRNA real-time PCR using U6 snRNA for an internal control (TIF) [file pone.0154942.s003.tif]

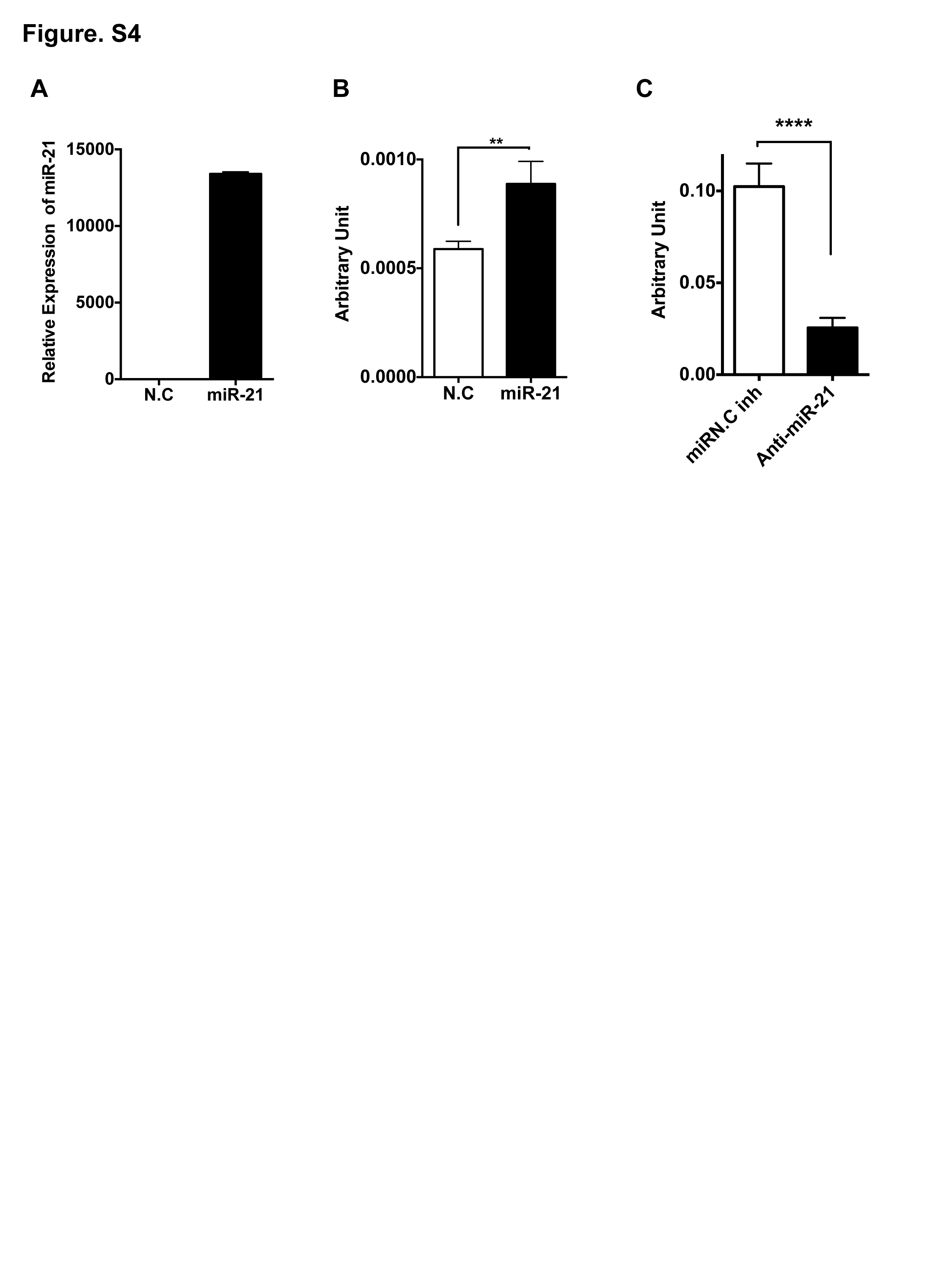

Supplement: S4 Fig — (A) The level of miR-21 was determined after transfection with hsa-miR-21 mimic (miR-21). (B and C) Luciferase activity of SBE (Smad Binding Element) after ectopic expression (B) or inhibition (C) of miR-21 was determined and shown as a bar graph. (B-C) These results represented one of the experiments performed twice with triplicate samples. (TIF) [file pone.0154942.s004.tif]
