# Supplementary material for: Induction of MiR-21 by Stereotactic Body Radiotherapy Contributes to the Pulmonary Fibrotic Response
Source: PLoS One. 2016 May 12;11(5):e0154942. doi: 10.1371/journal.pone.0154942 (PMC4865046; doi:10.1371/journal.pone.0154942)
Supplement: S1 File — (DOCX) [file pone.0154942.s005.docx]

**Supplementary Materials and Methods**

**Immunoblotting**

The cells were washed twice in ice-cold phosphate buffered saline (PBS), lysed with appropriate amount of tissue lysis buffer, stayed on ice at least 30 min and centrifuged for 10 min at 13,000 rpm (to clarify the lysates). 20-30 µg of total proteins were separated via SDS–PAGE. The proteins were then transferred to PVDF membranes, blocked for 1 h with 5% nonfat dry milk in Tris-buffered saline (TBS) with 0.05% Tween-20 (TBS-T), and incubated O/N with the appropriate primary antibodies in TBS containing 1% BSA solution on 4°C. The membranes were subsequently washed several times in TBS-T solution and incubated with HRP-conjugated secondary antibodies (0.1 µg/ml; Jackson ImmunoResearch Laboratories, West Grove, PA). Immunoreactivity was detected using an enhanced chemiluminescence detection system (Amersham Biosciences, Piscataway, NJ).

**Immunohistochemistry (IHC)**

Mouse lungs were fixed in 4% paraformaldehyde in 0.1 M phosphate buffer (pH 7.4) and the tissue samples were embedded in paraffin. The paraffin sections were deparaffinized, rehydrated, and subjected to citrate-based antigen retrieval. The sections were stained with masson’s trichrome staining for detection of lung fibrosis.

**Dual luciferase assay**

Cells were transfected with SBE-Luc and pRL plasmid with lipofectamine 2000 transfection reagent. At 24 hours after transfection, dual luciferase assay were performed according to manufacturer’s instruction (Promega), Promoter activity was normalized by activity of renilla.

**Supplementary Figure legends**

**S1 Fig. Induction of Collagen expression in SBRT induced lung injury** (A) The mRNA level of Collagen types (Col1A1, Col1A2, Col3A1, Col11A1 and Col15A1) of 2 and 4 weeks after 90Gy IR exposure was determined by real time PCR. Control indicated right side of the lung, which was not irradiated (n=2). 18s ribosomal RNA was used as a loading control.

**S2 Fig. Expression of a set of miRs was altered after local exposure of IR in the lung.** (A) Scatter plot of normalized intensity of miRs between control and IR damaged lung tissues at three weeks (left) and four weeks (right) (B) Heat-map of miRs (cut off range = 1.8) significantly altered in the IR damaged lung tissue compared to the control at both 3 (3W) and 4 (4W) weeks. Red dotted line and asterisk for significantly upregulated microRNAs after IR. (C) IPA of altered miRs at 3 or 4 weeks of IR damaged lung tissue

**S3 Fig.** **Increased levels of miR-21 are concurrent with IR-induced EndMT.** HPECs were exposed with 5 Gy of X-ray and were harvested at indicative time. Each results represent one of the experiments conducted three times with duplicate samples. (A) The mRNA levels of Col1A2, Col3A1 and FN at indicative time after 5 Gy by real-time PCR analysis, β-actin for an equal loading control. (B) The level of miR-21 at indicative time after 5 Gy by taqman miRNA real-time PCR using U6 snRNA for an internal control

**S4 Fig. miR-21 controls Smad dependent gene response through Smad7. (**A) The level of miR-21 was determined after transfection with hsa-miR-21 mimic (miR-21). (B and C) Luciferase activity of SBE (Smad Binding Element) after ectopic expression (B) or inhibition (C) of miR-21 was determined and shown as a bar graph. (B-C) These results represented one of the experiments performed twice with triplicate samples.
